# Spin-to-Charge Conversion in Orthorhombic RhSi Crystalline Thin Films

**DOI:** 10.1021/acsami.5c01170

**Published:** 2025-04-14

**Authors:** Surya N. Panda, Qun Yang, Darius Pohl, Hua Lv, Iñigo Robredo, Rebeca Ibarra, Alexander Tahn, Bernd Rellinghaus, Yan Sun, Binghai Yan, Anastasios Markou, Edouard Lesne, Claudia Felser

**Affiliations:** † Max Planck Institute for Chemical Physics of Solids, Nöthnitzer Str. 40, Dresden 01187, Germany; ‡ College of Letters and Science, University of California, Los Angeles, California 90095, United States; § Dresden Center for Nanoanalysis (DCN), Center for Advancing Electronics Dresden (CFAED), TUD Dresden University of Technology, Dresden D-01062, Germany; ∥ Institute of Metal Research, Chinese Academy of Science, Shenyang, Liaoning 110016, China; ⊥ Department of Condensed Matter Physics, Weizmann Institute of Science, Rehovot 7610001, Israel; # Physics Department, University of Ioannina, Ioannina 45110, Greece

**Keywords:** spin-pumping, ferromagnetic resonance, inverse spin Hall effect, epitaxial thin films, spin Berry curvature

## Abstract

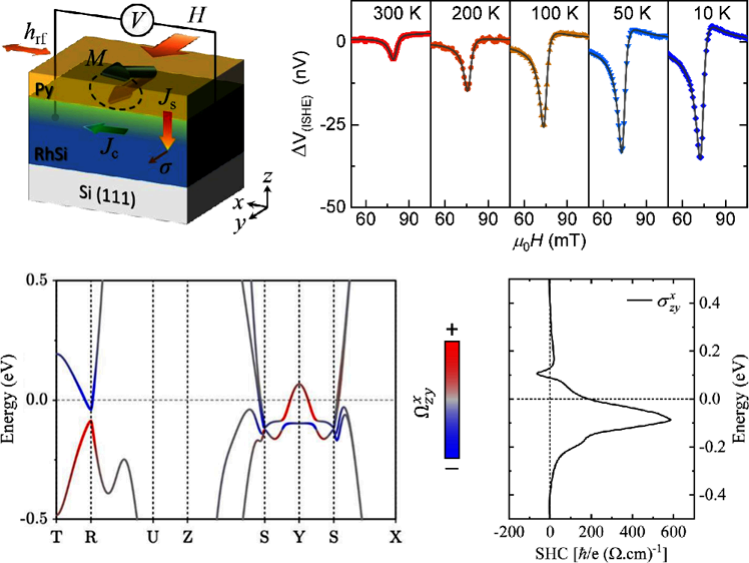

The rise of nonmagnetic topological semimetals, which provide a promising platform for observing and controlling various spin–orbit effects, has led to significant advancements in the field of topological spintronics. RhSi exists in two distinct polymorphs: cubic and orthorhombic crystal structures. The noncentrosymmetric B20 cubic structure has been extensively studied in the bulk for hosting unconventional multifold Fermions. In contrast, the orthorhombic structure, which crystallizes in the *Pnma* space group (No. 62), remains less explored and belongs to the family of topological Dirac semimetals. In this work, we investigate the structural, magnetic, and electrical properties of RhSi textured-epitaxial films grown on Si(111) substrates, which crystallize in the orthorhombic structure. We investigate the efficiency of pure spin current transport across RhSi/permalloy interfaces and the subsequent spin-to-charge current conversion via inverse spin Hall effect measurements. The experimentally determined spin Hall conductivity in orthorhombic RhSi reaches a maximum value of 126  at 10 K, which aligns reasonably well with first-principles calculations that attribute the spin Hall effect in RhSi to the spin Berry curvature mechanism. Additionally, we demonstrate the ability to achieve a sizable spin-mixing conductance (34.7 nm^–2^) and an exceptionally high interfacial spin transparency of 88% in this heterostructure, underlining its potential for spin–orbit torque switching applications. Overall, this study broadens the scope of topological spintronics, emphasizing the controlled interfacial spin-transport processes and subsequent spin-to-charge conversion in a previously unexplored topological Dirac semimetal RhSi/ferromagnet heterostructure.

## 1 Introduction

Topological semimetals are a novel class of quantum materials characterized by topologically nontrivial band structures,^1^ hosting Dirac,^2^ Weyl,^3^ or unconventional multifold Fermions,^4^ which have been the focus of extensive research in recent years. These semimetals provide a promising alternative for efficient spin current generation in spin–orbit torque (SOT) devices due to their intrinsically large spin Hall and orbital Hall conductivities, which originate from Berry curvature effects.^5−11^ Within the broader category of topological semimetals, transition metal–metalloid compounds (e.g., RhSi, CoSi, PdGa, PtAl) have attracted significant attention for their ability to host unconventional chiral multifold Fermions^12−14^ when crystallized in the noncentrosymmetric cubic B20 structure with a chiral *P*2_1_3 space group (No. 198). This multifold Fermionic behavior emerges from topologically protected band crossings (with 4- and 6-fold degeneracies) at certain high-symmetry points in the Brillouin zone, giving rise to several remarkable effects that are otherwise absent in other topological quantum materials.^15−19^

RhSi is particularly notable because it also functions as a topological Dirac semimetal when it crystallizes in its orthorhombic rather than cubic structure. Geller et al. initially reported the cubic B20 crystal structure in RhSi-like transition metal–metalloid composite structures.^20^ However, subsequent investigations by Schubert et al.^21^ revealed that these compounds can also adopt a B31-type orthorhombic crystal structure with a *Pnma* space group (No. 62). Moreover, Mozaffari et al.^22^ uncovered topological characteristics in the electronic band structure of orthorhombic RhSi, including a symmetry-protected Dirac nodal line and symmetry-enforced Dirac nodes at the S-point of the Brillouin zone. These Dirac nodes, which can be revealed by external magnetic fields, lead to anomalies in magnetic torque.^22−24^ Despite these intriguing properties, driven by the topology of its electronic band structure, orthorhombic RhSi has not yet been explored for its potential in spin-charge current interconversion or in the broader field of topological spintronics. Among transition metal–metalloid Weyl semimetals (e.g., RhSi, CoSi, PdGa, PtGa, PtAl), spin-to-charge interconversion has only been studied in polycrystalline cubic B20 CoSi thin films, using spin Hall magnetoresistance and harmonic Hall voltage measurement techniques.^25^ However, detailed investigations to elucidate the magnitude of spin-mixing conductance, interfacial spin transparency, and the spin Hall angle in these B20-based heterostructures along with their temperature dependence are still lacking. Such studies would shed light on the microscopic mechanisms of spin relaxation and spin-to-charge interconversion.

**Figure 1:**
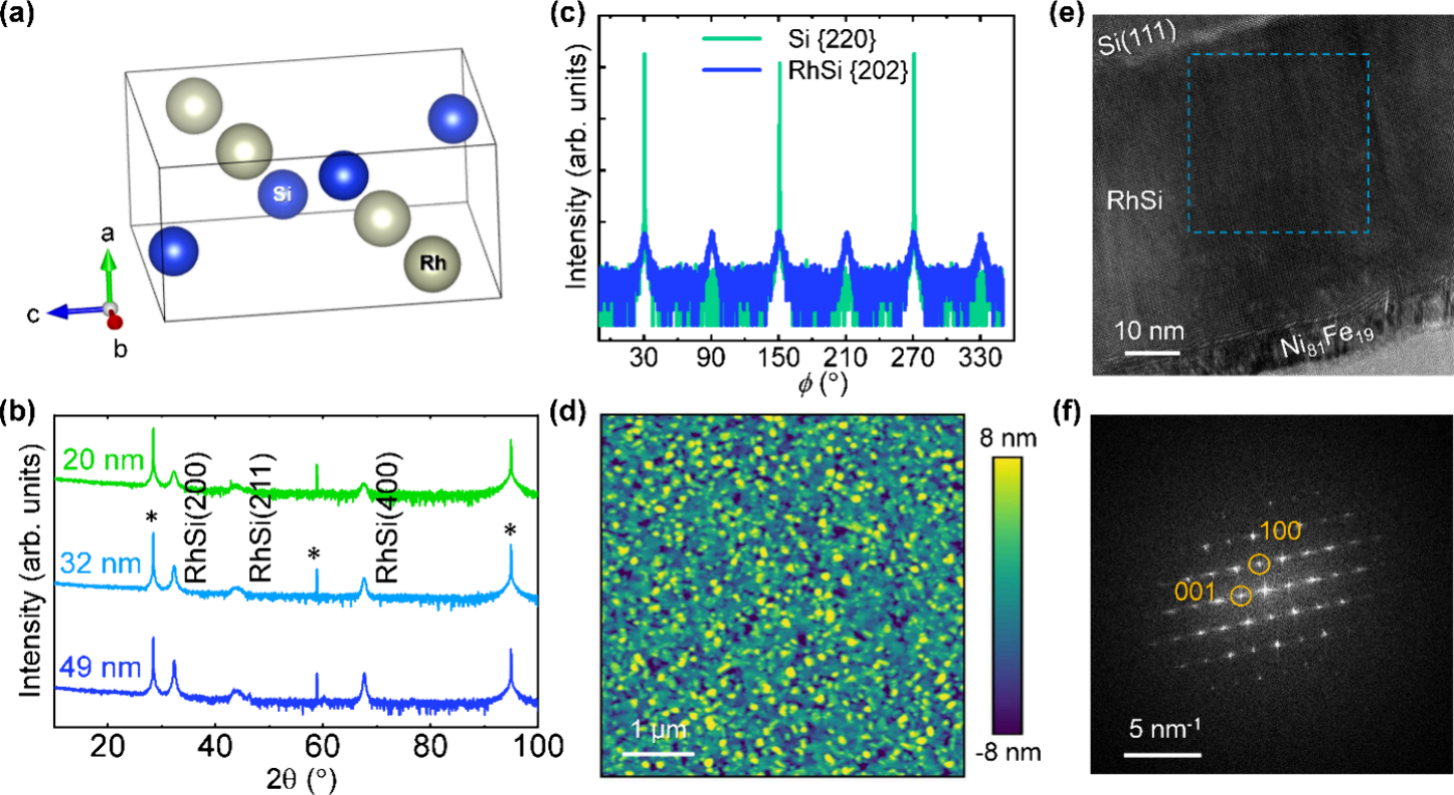
(a) Illustration of RhSi crystal structure that belongs to space group *Pnma* (No. 62). Gray and blue spheres denote Rh and Si atoms, respectively. (b) 2θ-ω XRD-pattern of RhSi thin films of different thicknesses grown on Si(111) single-crystal substrates (whose Bragg diffraction peaks are labeled by asterisks). (c) ϕ-scan patterns of {220} family of planes from a 49 nm-thick RhSi film and {120} family of planes from the Si substrate (d) AFM-image of an uncapped 25 nm-thick RhSi film showing surface topography. Color bar encodes height. (e) High-resolution TEM image of a 49 nm-thick RhSi/Py(6.6 nm) heterostructure acquired along the [010] zone axis of the RhSi film. Blue dashed square marks the area for fast Fourier transform analysis in panel (f). (f) FFT of RhSi film with identified reflections consistent with lattice spacings of orthorhombic space group *Pnma* (No. 62).

In this work, we investigate nonmagnetic topological semimetal RhSi/Ni_81_Fe_19_ (permalloy, hereafter Py) thin film heterostructures, grown via magnetron sputtering, using a combined ferromagnetic resonance (FMR)-driven spin-pumping generation and inverse spin Hall effect detection method. We experimentally determine the temperature dependence of the spin Hall angle (), spin diffusion length (), and spin Hall conductivity () in RhSi. Transmission electron microscopy and X-ray diffraction reveal that RhSi crystallizes in an orthorhombic structure with an achiral centrosymmetric *Pnma* space group (No. 62), as depicted in Figure 1a, which contradicts a previous report.^26^ We observe that lowering the temperature significantly enhances , , and , while also affecting spin-mixing conductance and interfacial spin transparency. From the temperature dependence of the effective Gilbert damping, we conclude that intraband conductivity-like scattering contributions dominate magnetization damping in Py thin films, while the enhanced Gilbert damping in RhSi/Py heterostructures is attributed to the spin-pumping mechanism. Furthermore, we examine the relative contributions of extrinsic mechanisms, such as spin-memory loss and two-magnon scattering, to the spin-pumping effect in RhSi/Py heterostructures.

## 2 Results and Discussions

### 2.1 Samples Characterization: X-Ray Diffraction (XRD)

The magnetron sputtering growth process of RhSi films and RhSi/Py heterostructures is detailed in the Methods section. The structure, crystallinity, and epitaxial relationship between the substrate and the sputter-deposited RhSi thin films were characterized using XRD measurements. Figure 1b shows 2θ-ω XRD scans of RhSi films with varying thicknesses (), each with a 3 nm-thick Si capping layer. In addition to the (111) and (222) reflections from the (111)-Si substrate, all samples exhibit the (200) and (400) reflections of orthorhombic B31-RhSi at 2θ ≈ 32.2° and 67.2°, respectively, indicating a preferred (100)-oriented growth of the RhSi films. We also observe a low-intensity broad peak at 2θ ≈ 46.4°, which corresponds to the (211) Bragg diffraction peak of orthorhombic RhSi. This finding contrasts with a previous report, which discussed RhSi films grown under similar conditions on Si(111) substrates as exhibiting a cubic B20-type structure.^26^ The interpretation of the XRD data is supported by transmission electron microscopy (TEM) refinements of the structure of our films, as discussed later. Furthermore, the confirmation of textured-epitaxial growth in these RhSi thin films is evidenced by the asymmetric XRD φ-scan (depicted in Figure 1c), which displays a 6-fold periodicity of the {202} family of planes that coincides in φ with the 3-fold periodic {220} Bragg family of planes in the Si substrate. This suggests the presence of twin domains in the RhSi films, further confirmed by TEM analysis.

### 2.2 Samples Characterization: Atomic Force Microscopy (AFM)

We conducted surface topography mapping of the RhSi films using AFM on replicas of films with nominally identical thicknesses as those studied in this research, but without a Si capping layer. AFM imaging was conducted immediately after the films were removed from the deposition chamber to avoid potential surface degradation from prolonged exposure to ambient air. In Figure 1d, an AFM-image of a 25 nm-thick RhSi film is presented, revealing an average root-mean-square roughness of 2.7 nm. Notably, the roughness was observed to increase slightly with increasing RhSi thickness. However, across the thickness range investigated in this study, no evidence of large-scale nonuniformity, discontinuity, or dislocations was detected.

### 2.3 Samples Characterization: Transmission Electron Microscopy (TEM)

To further support the XRD analysis of the crystalline structure of RhSi, we performed high-resolution TEM (HRTEM) imaging of a 49 nm-thick RhSi film interfaced with a permalloy overlayer (see Methods for details). Figure 1e shows the resulting HRTEM image, focusing on a contoured region of interest within the RhSi film, for which a fast Fourier transform (FFT) analysis has been postprocessed. The FFT image, presented in Figure 1f, confirms that RhSi grows textured in its orthorhombic crystal structure (space group No. 62), with the [100] direction (i.e., *a*-axis) aligned parallel to the [111] normal of the Si substrate. The detailed analysis of adjacent grains (see Supplementary Figure S1) reveals that they grow with either the [010] or [001] in-plane direction aligned parallel to the fixed [] zone axis of the Si substrate. This confirms the presence of twin domains in the RhSi films, with the *b*-axis and *c*-axis of the orthorhombic B31 structure lying within the plane of the film. Additionally, this allows us to determine the RhSi film’s lattice parameters *a* = 5.53 Å, *b* = 2.90 Å, and *c* = 6.49 Å.

### 2.4 Samples Characterization: DC Electrical Transport

We also conducted temperature-dependent measurements of the longitudinal resistivity of RhSi films using a van der Pauw configuration. Across all thicknesses investigated, metallic behavior was observed, characterized by a continuous decrease in longitudinal resistivity as the temperature decreased from 320 to 2 K. We found residual resistivity values as low as 55 μΩ·cm at 2 K, with minimal variation for films thicker than 20 nm (see Supplementary Figure S2). Notably, films of RhSi thinner than 19 nm exhibited higher resistivities and degraded metallic behavior, possibly due to surface and grain boundary scattering in the ultrathin limit.^27^ Therefore, we restricted our investigation to RhSi films thicker than 19 nm, which establishes an estimated thickness threshold for assessing the intrinsic electronic and spin-transport properties of these films.

### 2.5 FMR-Driven Spin-Pumping and Inverse Spin Hall Effect Measurements

To harness the unique properties of nonmagnetic topological semimetals in spin-based devices, it is necessary to investigate the efficiency of pure spin current generation and its subsequent transport across the interface with a ferromagnet (FM).^28^ Pure spin currents, which involve the flow of spins without any net charge flow, are critical for developing energy-efficient spin-based electronics, as they alleviate the limitations of charge-based devices, such as Joule heating and stray Oersted fields. Among the various mechanisms for generating spin currents,^29^ the “spin-pumping” phenomenon^30,31^ is particularly effective at generating pure spin currents at nonmagnetic (NM)/FM interfaces, as it avoids the well-known impedance mismatch problem.^32,33^ In the FMR-driven spin-pumping process, the precession of magnetization in the FM layer induces a finite electrochemical potential at the NM/FM interface, caused by the asymmetric accumulation of majority and minority spins. This nonequilibrium spin accumulation generates a pure spin current that diffuses into the NM layer, where it can undergo spin-to-charge current conversion, as spin angular momentum is not conserved. This diffusive spin current provides an additional channel for damping the out-of-equilibrium magnetization driven by FMR, typically leading to an increase in the FM layer’s Gilbert damping. The efficiency of the spin-pumping mechanism is primarily determined by the spin-mixing conductance (denoted ),^34^ while the magnitude of the injected spin current across the NM/FM interface is controlled by the interfacial spin transparency (denoted η).^35^

The FMR-driven spin-pumping approach has been extensively used to evaluate the primary spin-to-charge interconversion mechanisms and corresponding efficiencies,^36^ such as the inverse spin Hall effect (ISHE) and the associated spin Hall angle, and the inverse spin/orbital Rashba–Edelstein effect and corresponding Edelstein lengths. These processes convert a longitudinal spin current into a transverse electrical current.^37,38^ In the NM layer, the charge current resulting from ISHE produces a measurable voltage corresponding to the electromotive force, , which follows the relation^39^: , where  is the spin Hall angle of the NM material, and  is the spin-mixing conductance at the NM/FM interface. For pure spin current-based device applications, it is necessary not only to identify NM materials with a high spin Hall angle but also to engineer NM/FM heterointerfaces with high interfacial spin transparency and spin-mixing conductance.

**Figure 2:**
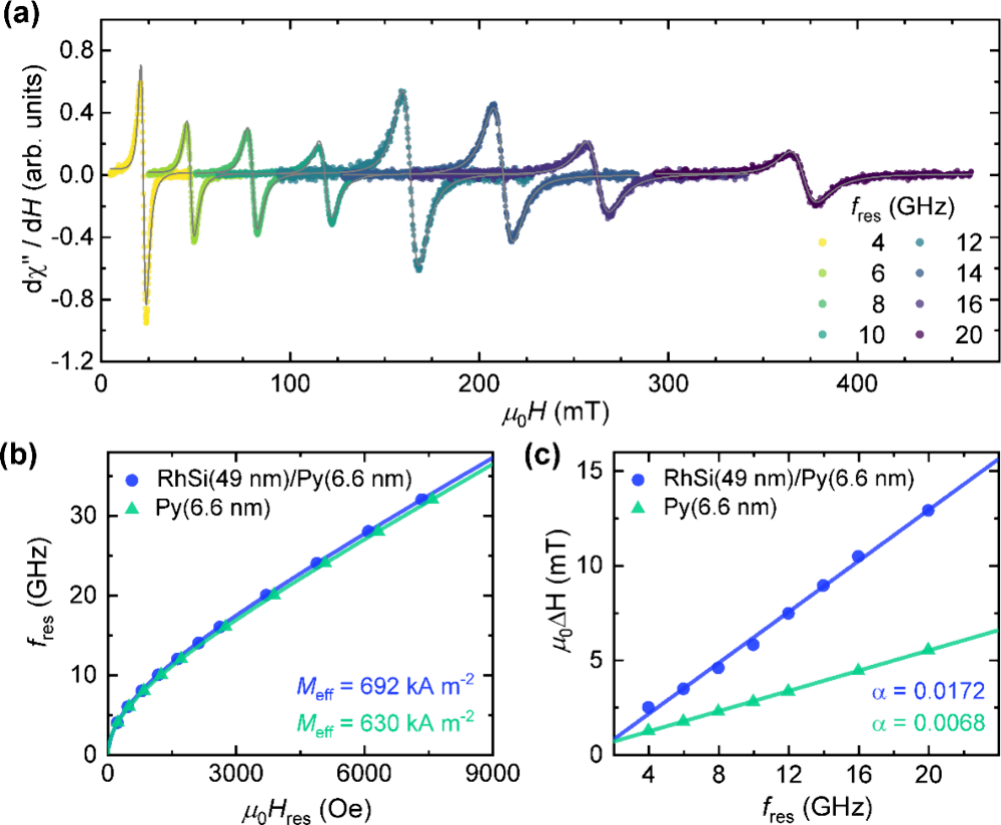
(a) FMR spectra of RhSi(49 nm)/Py(6.6 nm) sample measured at different resonant excitation frequencies (*f*_res_). The symbols correspond to experimental data and solid lines are the best fit using eq 1. (b) Field dependence of resonance frequency for RhSi(49 nm)/Py(6.6 nm) and Py(6.6 nm) samples. Solid lines are fitting results using Kittel’s formula, eq 2, which is parametrized by *M*_eff_. (c) Line width vs resonance frequency of FMR response. Solid lines indicate best fit using eq 4), whose slope corresponds to the Gilbert damping α.

In this study, the magnetization dynamics and the resulting Gilbert damping were measured using a NanOsc Instruments cryo-FMR setup (see Methods section). A typical FMR spectrum for a RhSi(49 nm)/Py(6.6 nm) sample measured at various excitation frequencies is shown in Figure 2a for the range of 4–20 GHz. The collected FMR spectra represent the field-derivative of the imaginary part of the dynamic magnetic susceptibility χ as a function of the applied field (*μ*_0_*H*). To determine the values of resonance field (*H*_res_) and line width (Δ*H*) from the FMR spectra, the data were fitted using the following formula^36^:
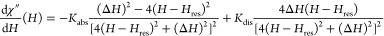
1where *K*_abs_ and *K*_dis_ are coefficients of the symmetric Lorentzian and antisymmetric components, corresponding to the absorption and dispersion contributions to the magnetic susceptibility, respectively. The effective saturation magnetization () and anisotropy field () are obtained from the dispersion relation of the resonance frequency vs field *f*_res_(*H*_res_), as described by Kittel’s formula^40^:
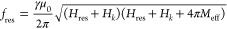
2where  is the gyromagnetic ratio,  and ℏ are the Bohr magneton and reduced Planck constant, respectively.  is the Landé -factor for the ferromagnetic layer. Using this fitting, *M*_eff_, , and  are determined as fitting parameters. Figure 2b shows the dispersion relation *f*_res_ vs  for the RhSi(49 nm)/Py(6.6 nm) and Py(6.6 nm) samples. The open symbols show the experimental data, while the solid lines represent the fit using eq 2). From these fits, we obtain values of *M*_eff_ ≈ 630 ± 8 kA m^–1^ in the presence of RhSi, and (692 ± 10) kA m^–1^ in its absence. This decrease in *M*_eff_ suggests a change in interfacial anisotropy, possibly due to modified interfacial spin–orbit interaction strength in the presence of RhSi.^41^ The effective electron -factor is found to be 2.11 ± 0.01 for both samples, a typical value for permalloy.^42^ In the absence and presence of RhSi the anisotropy field (*H*_*k*_) value is 2.1 and 1.1 mT, respectively.

The overall magnetization dynamics in the presence of spin pumping can be described by a modified version of the Landau–Lifshitz–Gilbert equation, given by^30,31^
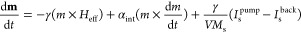
3where *V* is the volume of the FM, *M*_s_ is its saturation magnetization, *I*_s_^pump^ is the spin current injected into the NM layer, and *I*_s_^back^ is the backflow spin current returning to the FM layer. *m* denotes the magnetization () unit vector, i.e., . The net spin angular momentum flow across the NM/FM interface is determined by the balance between *I*_s_^pump^ and *I*_s_^back^.

The Gilbert damping parameter (α) is calculated by fitting the -dependent Δ*H* using the relationship^43^:
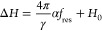
4where  denotes the frequency-independent line width broadening, which depends on magnetic inhomogeneities within the sample. The effective Gilbert damping parameter α obtained from this analysis includes contributions from intrinsic spin-pumping and extrinsic effects including spin-memory loss, two-magnon scattering, interfacial band hybridization, and magnetic proximity effects. Figure 2c shows the  vs  dispersion relation for the RhSi(49.1 nm)/Py(6.6 nm) and Py(6.6 nm) samples. The linear behavior of Δ*H*() implies good homogeneity in our samples.  value is found to be less than 1 mT in both the presence and absence of the RhSi underlayer. The determined value of α for RhSi(49.1 nm)/Py(6.6 nm) is 0.0172 ± 0.0005, which is significantly higher than the Gilbert damping parameter for the Py(6.6 nm) reference sample 0.0068 ± 0.0004. This difference is consistent with the presence of an efficient spin-pumping effect, as discussed further. However, it is important to note that other extrinsic factors may also contribute to the observed enhancement in α.

The transfer of spin angular momentum across the NM/FM interface, without any spin backflow effect, is characterized by the intrinsic spin-mixing conductance, denoted as . This intrinsic  represents the conductance properties of spin channels at the NM/FM interface when the NM layer thickness () is much greater than the spin diffusion length (), which represents the characteristic distance traveled by spin currents before dissipation in the NM layer. In the presence of spin backflow (where  ≤ ), spin transport through the interfacial spin channels is described by an effective spin-mixing conductance (), which depends on the material properties, the interface, and the thickness of the NM layer. The flow of spin angular momentum across the interface exerts a damping-like torque on the magnetization, leading to an increase in the Gilbert damping parameter. The modulation of the Gilbert damping parameter, ), can be related to the spin-mixing conductance as follows^36,44^:
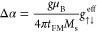
5where  is the thickness of the FM layer. In the presence of the spin-pumping effect, α increases nonmonotonically and saturates as the thickness of the NM layer increases. It is important to note that this description of the spin-pumping effect assumes a constant and thickness-independent resistivity of the NM layer. However, the effective Gilbert damping in NM/FM can be affected sensitively by changes in resistivity of either or both the NM and FM layers.^45,46^ In this study, we have taken care of maintaining the thickness and resistivity of the Py layer constant, and have carefully measured the temperature-dependent resistivity of our RhSi films across the entire thickness series. Therefore, for films thicker than 19 nm, as studied here, the observed enhancements in  primarily reflect the efficiency of the spin-pumping effect. Some studies^47^ have indicated that thermal gradients in thin film heterostructures can lead to spin Seebeck effect and anomalous Nernst effect. However, in Py-based systems, and for spatially extended blanket films of several mm^2^ studied here (see Methods), such prospective thermal gradients are very small,^48^ and the temperature elevation of the sample at resonance negligible (of the order of hundreds of mK). Thus, the aforementioned thermally driven effects are not expected to contribute relevantly to the overall spin-pumping signal.

### 2.6 ISHE Measurements and Determination of Spin Hall Conductivity

**Figure 3:**
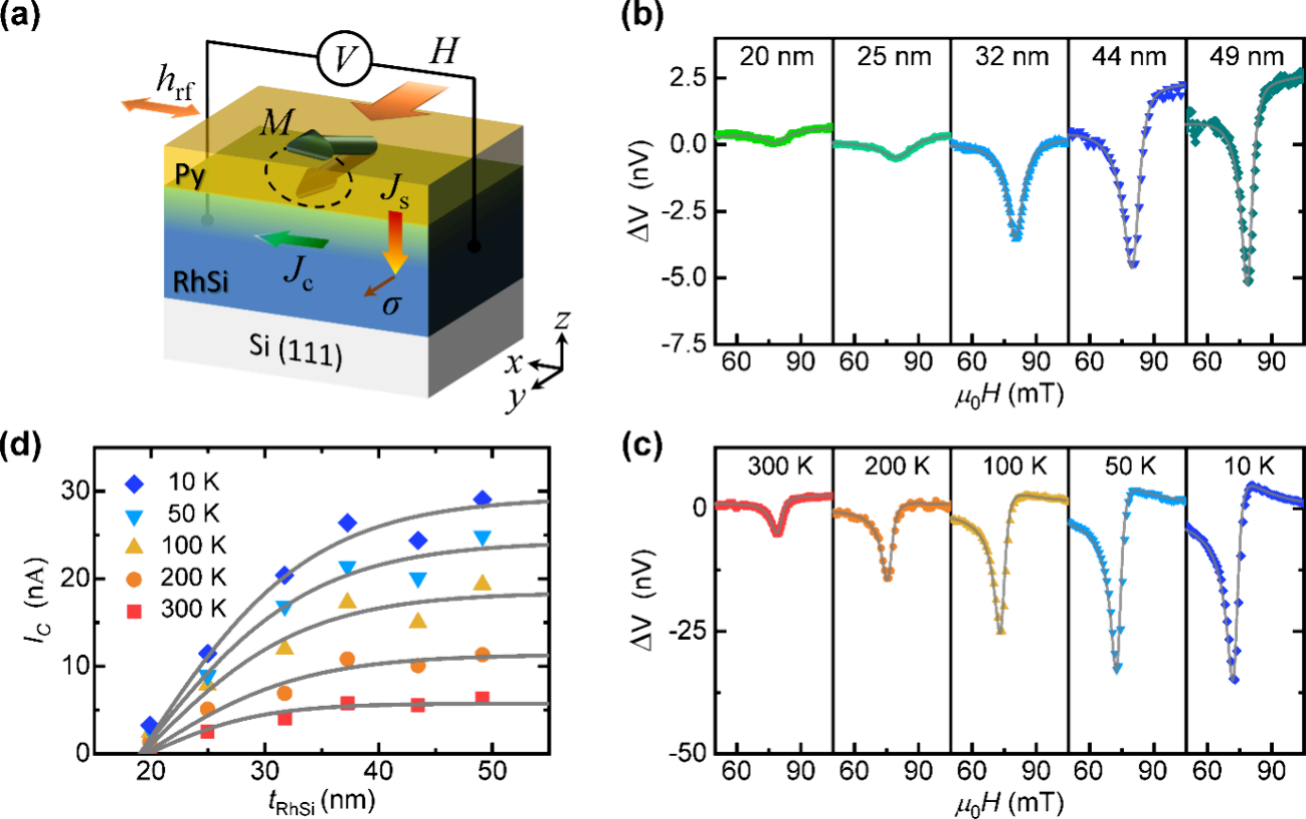
(a) Schematic of sample structure and measurement geometry for FMR-driven spin pumping and inverse spin Hall effect (ISHE) measurements. Voltage drop detected in FMR-spin pumping experiments at 8 GHz (b) for RhSi(*t*_RhSi_)/Py(6.6 nm) heterostructures at 300 K, and (c) at various temperatures (10–300 K) for a 49 nm-thick RhSi/Py(6.6 nm) sample. (d) RhSi-thickness-dependent modulation of ISHE-induced charge current fitted using eq 7) (solid red lines), at various temperatures.

In NM materials exhibiting a sizable SHE, an efficient spin-pumping effect is accompanied by a characteristic voltage drop,  due to the ISHE.^37^ This effect causes the generated spin current density, , to be converted into a transverse charge current density,  (or a DC voltage in an open circuit), within the high SOC material. The efficiency of this interconversion mechanism is parametrized by the spin Hall angle θ_SH_ = . The schematic experimental geometry of the spin-pumping and combined ISHE measurement configuration is shown in Figure 3a; see the Methods section for further details. The overall voltage drop (denoted Δ*V*) is composed of both field-symmetric and antisymmetric components (see Supplementary Figure S3), which can be separated by fitting the experimental data with a combination of (anti)Lorentzian functions^49,50^:
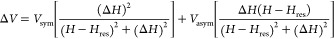
6where  is attributed to the ISHE contribution,^30,31,36^ and  represents contributions from other extrinsic effects, such as rectification.^51^ The generated charge current, *I*_c_, within the RhSi layer is given by the ratio between *V*_ISHE_ and the sheet resistance of the RhSi film, ). Figure 3b displays the measured voltage drop minus an offset voltage (), at 300 K for a series of RhSi films with various thicknesses (ranging from 20 to 49 nm), measured during FMR-spin pumping and ISHE experiments conducted at 8 GHz. Additionally, the temperature dependence of  is shown in Figure 3c, where the overall amplitude of  monotonically increases as the system temperature decreases from 300 to 10 K.

In Figure 3d, we have plotted the RhSi thickness dependence of the ISHE-induced charge current *I*_c_ at different temperatures. We observe that *I*_c_ increases monotonically with increasing RhSi thickness and eventually saturates at higher thicknesses, in agreement with eq 7) under the ISHE mechanism. Additionally, the magnitude of *I*_c_ systematically increases as temperature decreases, indicating enhanced spin-to-charge interconversion efficiency at lower temperatures in our RhSi thin films, as discussed in the next section.

The reported RhSi thickness-dependent *I*_c_ can be used to extract the characteristic spin Hall angle () and spin diffusion length () of RhSi, which are related by the following expression^52,53^:
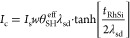
7where , the spin current generated by spin pumping, is given by
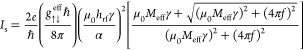
8

 is the width of the sample, and  is the magnitude of the radiofrequency (RF) magnetic field. According to the calibration (see Methods),  ≈ 60 μT. We denote  the effective SH angle extracted using this formalism. As will be discussed later, the estimation of an intrinsic  value requires considering additional mechanisms (e.g., interfacial spin transparency), which can affect its magnitude and cause  to differ from the measured . The behavior of the measured *I*_c_ vs *t*_RhSi_ is well described by eq 7) at all measurement temperatures, as shown by the solid red lines in Figure 3d. The value of , extracted from eq 5, is used in eq 8 to extract the magnitude of the spin current density *J*_s_ = *I*_s_/(*t*_RhSi_·*w*) at each temperature.

**Figure 4:**
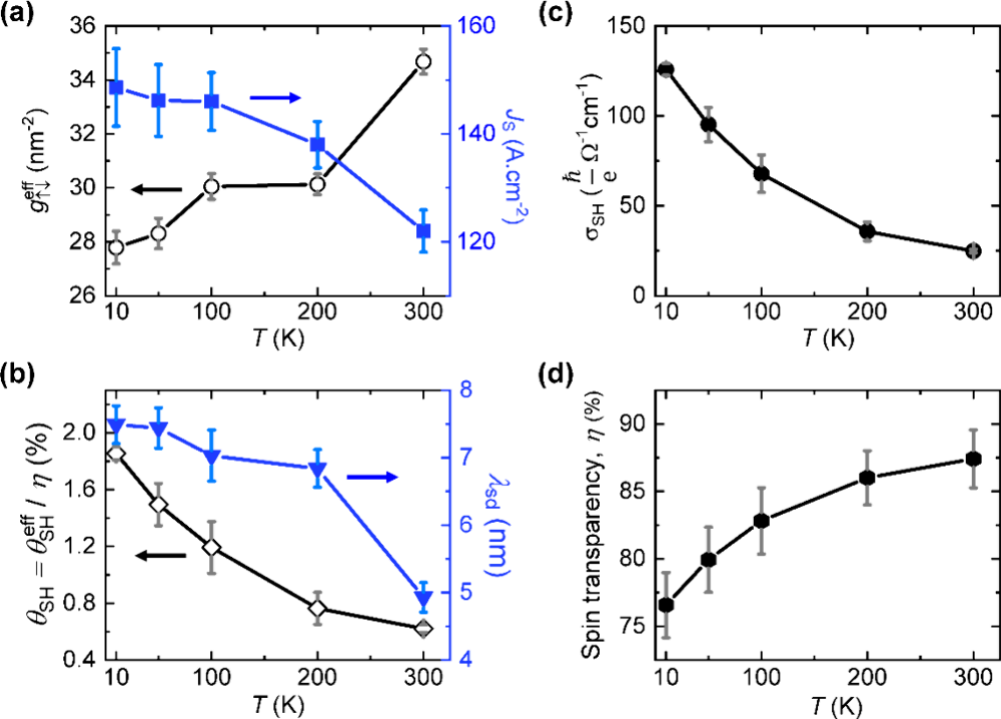
Temperature-dependent variation of (a) effective spin-mixing conductance (left axis) and generated charge current via spin pumping (right axis), (b) spin Hall angle (left) and spin diffusion length (right) of RhSi, (c) spin Hall conductivity of orthorhombic RhSi films, and (d) interfacial spin transparency of RhSi/Py heterostructures.

Figure 4a shows the variation of *J*_s_ and  with temperature. We observe that *J*_s_ decreases whereas  increases with increasing temperatures. The decrease in *J*_s_ can be attributed to increased dissipation of accumulated spin densities at the interface due to both bulk and interfacial spin scattering, which also leads to the observed increase in .^54^ The rise in  with temperature signifies a higher probability of spin transfer and increased losses of spin angular momentum from the FM at elevated temperatures. This trend in  also indicates that interfacial spin accumulation decreases with increasing temperature facilitating a more efficient spin transfer between Py and RhSi at higher temperatures. This trend in  could be further attributed to the presence of some level of interfacial disorder (both structural and electronic) whose impact is more pronounced at lower temperature and which consequently reduces the spin-mixing conductance in our Py/RhSi samples. Figure 4b shows the temperature dependence of  and  extracted from the fitting of *I*_c_ vs *t*_RhSi_ in Figure 3d. We chose to display  as the measured effective spin Hall angle, corrected by the estimated value of the interfacial spin transparency η (as discussed in the following section). Regardless, we observe that both  and  decrease with increasing temperatures. The decrease in  with temperature is more straightforward to understand and is expected when the Elliott–Yafet (EY) scattering mechanism dominates the spin relaxation process. Within the EY mechanism, the spin scattering probability scales with the momentum relaxation rate,^55^ leading to , where  is the longitudinal resistivity of the RhSi films. We measured = 109.2 μΩ.cm (56.7 μΩ.cm) at 300 K (10 K), corresponding to  4.9 nm (7.5 nm). This shorter  at higher temperatures is also due to higher spin resistance (), where *V* is the volume in which the spin current diffuses, as predicted by the spin diffusion model.^56^ The temperature dependence of  and the spin Hall conductivity (SHC), , in RhSi clarifies the mechanisms contributing to the ISHE in our heterostructures. Notably,  decreases from 1.8% at 10 K to a moderate 0.6% at RT, a behavior similar to that observed in Au films,^56^ which are known to exhibit very low intrinsic SHC.

**Figure 5:**
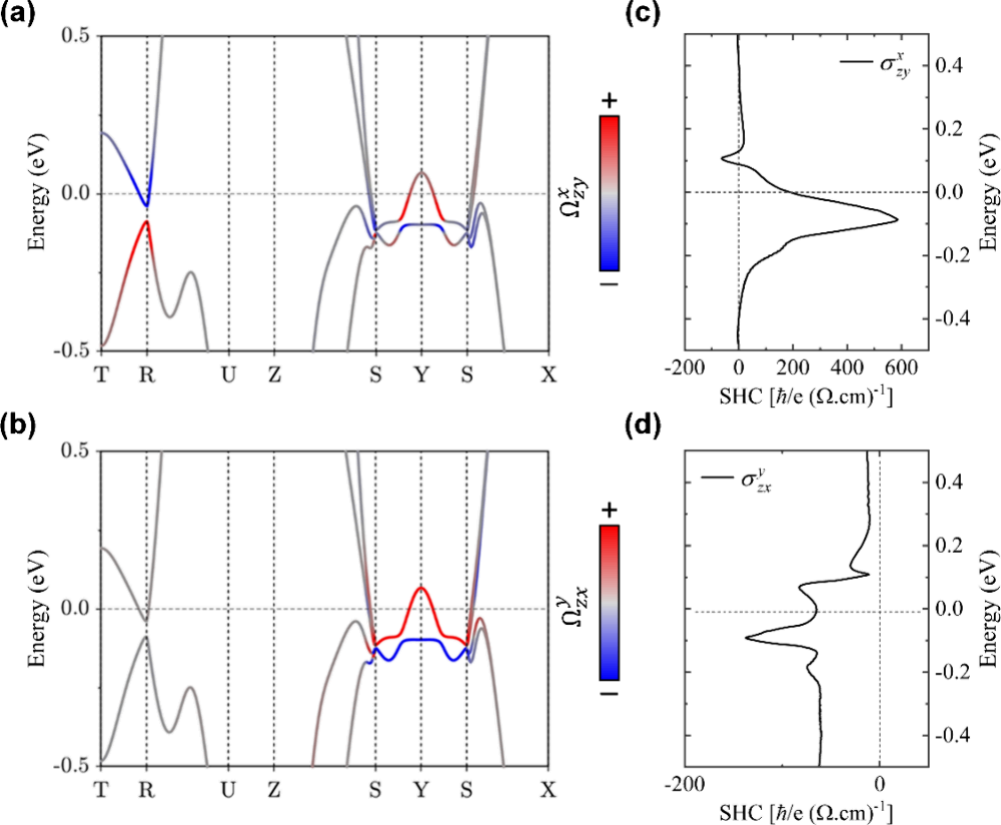
Spin Berry curvature resolved band structure along high-symmetry lines of the BZ for (a)  and (b)  of orthorhombic RhSi. Red (blue) denotes positive (negative) contributions. (c, d) Corresponding energy-dependent *k*-integrated SHC tensor elements accessible experimentally.

Furthermore, it is possible to estimate the SHC of RhSi films. Under the condition where the longitudinal resistivity , the SHC is given by . The temperature dependence of  is displayed in Figure 4c. The value of  decreases from 106  at 10 K to 25  as the temperature increases to 300 K, as shown in Figure 5c. The total , is characteristically the sum of intrinsic and extrinsic contributions, acting as parallel channels (). The intrinsic , originating from the spin Berry curvature mechanism, is temperature-independent because it does not rely on the momentum relaxation time and thus is not expected to depend on ρ_RhSi_. However, the extrinsic term  originates from various extrinsic scattering mechanisms, such as skew scattering at impurities and phonons, or side-jump scattering in highly resistive materials. This extrinsic contribution is expected to display a temperature dependence that reflects its multiple originating mechanisms. Although we cannot entirely rule out these contributions to the measured signal, assessing their individual contributions remains challenging. For these reasons, in a following section, we rather decide to focus the discussion on the intrinsic Berry curvature source of SHE in RhSi in the context of first-principles calculations.

### 2.7 Role of Interfacial Spin Transparency in Spin Transport across RhSi/Py Interfaces

The generation of a pure spin current through spin pumping does not necessarily ensure that all the spins accumulating at the NM/FM interface will successfully diffuse through the NM layer and undergo spin-to-charge conversion therein. Factors such as electronic band alignment, disorder, and intermixing at the NM/FM interface play a crucial role in determining the probability of spin transmission across the interface. The concept of interfacial spin transparency, denoted η, accounts for all these effects, which influence whether electrons are reflected from the interface or transmitted during spin transport. In the diffusive spin-transport model, η can be expressed as a function of  and  using the following relation^35,57^:
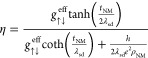
9where  is the resistivity of the NM layer, *h* is Plank’s constant, and *e* is the elementary charge.

The magnitude of η provides a qualitative measure of the spin transparency at the RhSi/Py interfaces. Recent studies suggest that in NM/FM heterostructures, both  and the strength of the SOT are dependent on the precise value of η.^35,58^ In Figure 4d, we display the η-value for RhSi/Py heterointerfaces, which shows a monotonic increase with rising temperature. The spin transparency remains consistently high, varying from 77 at 10 K to 88% at 300 K. This increase in interfacial spin transparency η may be attributed to improved electronic band matching at higher temperatures and the activation of thermally activated spin-transport mechanisms across the interface. Consequently, the spin transport becomes less sensitive to local disorder or imperfections that could otherwise hinder interfacial spin transmission.

We stress out that the specific temperature dependence of each spin-dependent quantity reported in Figure 4 cannot be easily attributed uniquely to one given microscopic mechanism, nor can we exclude some level of extrinsic contribution, as mentioned earlier. This includes spin-dependent scattering at defects and impurities and their rate of change due to temperature-dependent electron–phonon interactions or screening effects. Additional contributions due to thermal expansion (and their mismatch at interfaces), changes in lattice parameters and their impact on strain and the electronic band structure (of both RhSi and the spin-active interfacial region), as well as on the position of the Fermi level are beyond the scope of the present experimental investigation, but could warrant further attention.

### 2.8 Electronic Band Structure and SHC Calculations

To gain insights into the intrinsic origin of spin-to-charge interconversion in RhSi, which is driven by the spin Berry curvature, we performed density functional theory (DFT) calculations using the Vienna Ab initio simulation package for orthorhombic RhSi (space group No. 62),^59,60^ with the experimentally determined lattice parameter values. Within the framework of the SHE, the externally applied electric field along the β-direction () and the induced spin current along the α-direction, with spin polarization along the γ-direction (), are related by the SHC tensor () as =. We reframe (α,β,γ) to align with the natural coordinate system (*x*, *y*, *z*), constrained by the experimental geometry and the preferential *a*-axis texture of the RhSi films (see Figure 3a). In this system, the *z*-axis aligns with the *a*-axis of RhSi (which also corresponds to the direction of the spin current density, ), whereas the *x*- and *y*-axes lie within the film plane, corresponding to the *c*- and *b*-axis directions of RhSi, respectively. Thus, the SHC tensor elements  and  are the relevant components accessible through the experimental technique and geometry (see Table 1). We calculated  and  to be −65 and 190 , respectively, at the Fermi level.

**Table 1:** Calculated Spin Hall Conductivity of Orthorhombic RhSi (*Pnma* space group No. 62) at the Fermi Level^a^

	σ^*x*^	σ^*y*^	σ^*z*^
6 independent SHC tensor components	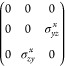	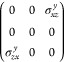	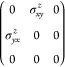
*z* ≡ [100]	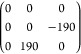	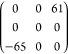	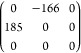

a SHC magnitude in units of . *z*-direction (*x* and *y*) corresponds to [100] axis (in-plane [001] and [010]) of RhSi.

Notably, the experimental value of  at 10 K (126 ) closely matches the average of these two inequivalent SHC tensor components. This agreement is consistent with the presence of twin domains oriented in-plane along either the [001] or [010] principal axis directions of RhSi, as supported by XRD and TEM characterizations (see Figure 1 and Supplementary Figure S1). Beyond the quantitative agreement between experiment and theory, the sign and magnitude of the measured SHC are fully consistent with expectations from an intrinsic spin Berry curvature mechanism for a twinned (100)-textured RhSi orthorhombic crystal.

We calculated the *k*-resolved spin Berry curvature of the band structure and the energy-dependent SHC. As shown in Figure 5a,b, the red and blue color-coded scales denote significant positive and negative contributions to the spin Berry curvature, respectively. Orthorhombic B31-RhSi exhibits topological features with multiple Dirac nodes and a symmetry-protected Dirac nodal line in its band structure.^22^ Specifically, the band structure reveals multiple band crossings along high-symmetry (HS) lines, starting from the HS point *S* in its 3D Brillouin Zone (BZ). Additionally, symmetry-protected band crossings occur along the SX lines near the Fermi energy, leading to a symmetry-protected nodal line around the *S*-point on the  plane. Moreover, the SY, TR, and RU HS lines are identified as 4-fold-degenerate HS lines enforced by the crystal symmetries. As shown clearly in Figure 5a,b, these 4-fold-degenerate HS lines serve as sources of substantial spin Berry curvature distributions  and . Moreover, because the SHC depends on the Fermi level, it varies rapidly when the Fermi energy shifts by only a few tens of meV. Figure 5c,d illustrate that both  and  exhibit peaks of 587 and −137 , respectively, when the Fermi energy is positioned at approximately −0.1 eV, which is near the band crossing points along the SX lines. This energy-dependent analysis indicates a potential strategy for enhancing the SHE in RhSi by fine-tuning the Fermi level position, which could be achieved through techniques such as epitaxial strain, external pressure, substitutional doping or electrostatic gating.

## 3 Conclusions

In summary, we investigated spin pumping and the ISHE in textured-epitaxial (100)-RhSi topological semimetal thin films, grown on Si(111) substrates, which crystallized in the orthorhombic structure with the *Pnma* space group No. 62. We demonstrated that, in the presence of RhSi, the magnetization dynamics are dominated by the spin-pumping effect, which can be adjusted by changing the thickness of RhSi and Py. We evaluated the Gilbert damping parameter, spin-mixing conductance, interfacial spin transparency, spin Hall angle, and SHC for the RhSi/Py system by modeling the experimental results. Additionally, we achieved broad tunability of these parameters with system temperature and RhSi thickness. At the lowest accessible temperature (10 K), RhSi/Py heterostructures displayed a maximum spin Hall angle of 1.8% for RhSi, a spin-mixing conductance of 34.7 nm^–2^, and an interfacial spin transparency of 88%. The substantial intrinsic SHC, along with the high interfacial spin transparency in RhSi/Py, suggest that orthorhombic RhSi is a promising material for pure spin current-based spintronics and spin–orbitronics applications. The values of  and η for the RhSi/Py interface are significantly higher than those of the widely used Pt/Py interface and compare positively with a number of other canonical (e.g., Pt/YIG) and more exotic NM/FM heterostructures, such as incorporating topological insulators, van der Waals layered transition metal dichalcogenides or Heusler alloys, and frequently studied in spintronic devices (as summarized in Table 2). This further confirms that RhSi serves as an excellent spin sink, resulting in a robust spin pumping effect. The temperature dependence of η, , , *M*_eff_, α, , and  provides essential guidance for selecting the appropriate material combination for spintronics devices with desired operating conditions. However, for spin-based device applications, these parameters can be further optimized by selecting different NM topological semimetals, engineering interfacial layers (e.g., to tailor Rashba spin–orbit coupling,^61^ or enhance interfacial spin transparency), and adjusting their thicknesses, in conjunction with a chosen FM layer. Therefore, the parameter space for enhancing the functionalities and performance of topological semimetal/FM interfaces for spintronics remains largely unexplored.

**Table 2:** Comparison of the (Effective) Spin-Mixing Conductance (), Spin Diffusion Length (), Spin Hall Angle (), and Interfacial Spin Transparency (η) of the RhSi/Py Samples Studied Here with Selected Values at Spin Hall Material/FM Interfaces Taken from the Literature^a^

heterostructures	(nm^–2^)	(nm)	(%)	η
RhSi/Py (this work)	34.7	7.5	1.8	0.88
Pt/Py^37^	15.2	1.4	19	0.25
Pd/Py^65^	14.0	5.8	N.A.	N.A.
Au/YIG^66^	2.7	60	8.4	N.A.
Ag/YIG^66^	5.2	700	0.68	N.A.
W/YIG^66^	4.5	2.1	14	N.A.
Ta/YIG^66^	5.4	1.9	7.1	N.A.
Pt/YIG^66^	6.9	7.3	10	N.A.
Bi_2_Se_3_/CoFeB^67^	12	5.0	43	N.A.
CoSi/CoFeB^25^	N.A.	4.4	3.4	N.A.
MoS_2_/CoFeB^68^	14.3	7.83	N.A.	N.A.
Pt/Co_2_FeAl_0.5_Si_0.5_^51^	3.7	3.06	1.6	N.A.

a N.A. stands for data not available.

Our findings may pave the way for the development of spin–orbitronics devices based on newly synthesized topological Dirac semimetals in thin film form, especially in applications such as SOT magnetic random-access memories, where η, , and α play a critical role in enhancing switching speed and overall efficiency. We believe that various strategies, such choosing specific combinations of FM and NM materials, electrostatic field effect, strain, or tuning materials’ composition and Fermi level via chemical doping, could enhance the intrinsic SHC, improve interfacial spin current transport, as well as increase the overall efficiency of spin-to-charge current conversion. Furthermore, disentangling the contributions of the putative orbital Hall effect^62,63^ where finite spin–orbit interaction is not required from the overall transverse signal attributed to the SHE opens up new possibilities in the emerging field of orbitronics, potentially allowing the generation and propagation of orbital rather than spin information over distances far exceeding characteristic spin diffusion lengths.^64^

## 4 Methods

### 4.1 Magnetron Sputtering Growth

Thin films of RhSi()/Py()/SiO_*x*_(3 nm) were grown employing a BESTEC UHV magnetron sputtering system on (111)-oriented Si single-crystal substrates. The thickness of the RhSi layer was varied from 19 to 55 nm, and the Py layer thickness ranged from 6.6 to 30 nm. Before the deposition process began, the chamber was evacuated to a base pressure of less than 5 × 10^–9^ mbar, with the process gas (Ar 5 N) pressure set to 3 × 10^–3^ mbar. The target-to-substrate distance was maintained at 18.6 cm to ensure optimal sputtering conditions. To achieve spatial uniformity, the substrate was rotated at 24 rpm during deposition. The growth of (100)-oriented RhSi films was conducted at 680 °C, with Rh and Si sources in confocal geometry operated at 15 W DC power and 80 W RF power, respectively. Following the RhSi deposition, the ferromagnetic Py layer was deposited at room temperature using a DC power of 40 W applied to a Ni_81_Fe_19_ target. Each sample was capped in situ with a protective Si layer to prevent oxidation of the metallic silicide or permalloy films. The deposition conditions were carefully optimized and maintained consistently for all samples.

### 4.2 Characterizations

Symmetric and asymmetric X-ray diffraction (XRD) scans were carried out using a Panalytical XPert^3^ XRD diffractometer with Cu Kα_1_ radiation (λ = 1.5406 Å). The electron density, interface roughness, and layer thicknesses were determined through X-ray reflectivity measurements. Surface topography was analyzed using atomic force microscopy (AFM) with an MFP-3D Origin microscope from Asylum Research (Oxford Instruments). TEM was conducted with a JEOL JEM F200, operated at an acceleration voltage of 200 kV and equipped with a GATAN OneView CMOS camera for fast imaging. Local EDS analysis was performed using a dual 100 mm^2^ window-less silicon drift detector.

For longitudinal resistivity measurements, we employed a Physical Properties Measurement System (PPMS) Quantum Design cryostat and ultrasonically bonded aluminum wires to the corners of the square samples in a van der Pauw geometry. Frequency-dependent FMR measurements were conducted with a NanOsc FMR setup, integrated with a Quantum Design PPMS. The sample, typically 3 mm × 5 mm in size, was positioned in a flip-chip configuration atop a 200 μm wide coplanar waveguide (CPW). FMR measurements were conducted across frequencies ranging from 4 to 20 GHz (and up to 40 GHz), spanning temperatures from 10 to 300 K, in an in-plane geometry. As per the careful RF power vs frequency calibration provided by the FMR spectrometer instrument provider (NanOsc), the RF field magnitude is estimated to be 60 μT and nearly frequency-independent in the 4 to 20 GHz range used for this study. The magnetic field was swept across a predefined range while the frequency remained constant. To measure the ISHE, electrical contacts were established on opposite sides of the long edge of the sample using silver paste and platinum wires, and the ISH voltage was measured using a Keithley 2182A nanovoltmeter. We have also carefully verified and optimized the response of our cryogenic NanOsc FMR setup using standard Ni_81_Fe_19_/Pt thin film heterostructures, which yield values of spin-mixing conductance, spin Hall angle and spin diffusion length within the characteristically reported values in the literature for this specific benchmark system for spin-pumping/ISHE experiments.

### 4.3 First-Principles Calculations

The exchange-correlation potential was described using the generalized gradient approximation, following the Perdew–Burke–Ernzerhof parametrization scheme.^69^ A *k*-point grid of 8 × 8 × 8 was employed, and the total energy convergence criterion was set to  eV. From DFT calculations, we projected the ab initio DFT Bloch wave functions into highly symmetric atomic-orbital-like Wannier functions using a full-potential local-orbital minimum-basis code (FPLO).^70^ This allowed us to generate the corresponding tight-binding (TB) model Hamiltonian, which fully respects the symmetry of the materials under study. In the context of SHE, the applied electric field along the β-direction () and the induced spin current along the α-direction with spin polarization along the γ-direction () are related by the SHC tensor () as *=*. Using the obtained TB Hamiltonian, the intrinsic SHC tensor  was calculated via the Kubo formula:
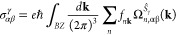
10
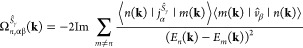
11where  is the conventional spin current operator, and  represents the spin Berry curvature.  is the spin operator,  is the eigenvalue for the  eigenstate  at momentum , and  is the ) component of the band velocity operator defined by , and  is the Fermi–Dirac distribution function. For the integration in eq 10), a uniform 240 × 240 × 240 *k*-grid was used to perform the *k*-space summation.

We aligned the *z*-axis with the crystallographic *a*-axis, corresponding to the experimental setup where the spin current flows along the *z*-axis (crystallographic *a*-axis) and the charge current flows along either the *x*- or *y*-axis. Orthorhombic RhSi belongs to the space group *Pnma*, which is defined by the 2-fold screw rotations { | , 0, }, { | 0, , 0} and inversion symmetry }. These symmetries combine to give three screw axis operations { | , 0, }, { | , , }, and { | 0, , 0}, as well as three glide plane operations { | , 0, }, { | , , }, and { | 0, , 0}. The material also exhibits time-reversal symmetry. Constrained by these symmetry operations, many SHC tensor elements are forced to zero, leaving only six independent nonzero elements, as shown in Table 1.

## Supporting Information Available

RhSi TEM grain analysis; longitudinal resistivity of RhSi crystalline thin films; temperature- and thickness-dependence of Gilbert damping parameter and saturation magnetization; permalloy thickness-dependent FMR; and symmetric and antisymmetric components of ISH voltage measurements (PDF)
