# Supplementary material for: Spin-to-Charge Conversion in Orthorhombic RhSi Crystalline Thin Films
Source: ACS Appl Mater Interfaces. 2025 Apr 14;17(16):24157–67. doi: 10.1021/acsami.5c01170 (PMC12022955; doi:10.1021/acsami.5c01170)
Supplement: Supplementary file 1 — am5c01170_si_001.pdf [file am5c01170_si_001.pdf]

# – Supporting Information –

Spin-to-Charge Conversion in Orthorhombic RhSi Crystalline Thin Films

Surya N. Panda[1*], Qun Yang[2], Darius Pohl[3], Hua Lv[1], Iñigo Robredo[1], Rebeca Ibarra[1], Alexander Tahn[3], Bernd Rellinghaus[3], Yan Sun[4], Binghai Yan[5], Anastasios Markou[1,6], Edouard Lesne[1*], and Claudia Felser[1*]

[1]Max Planck Institute for Chemical Physics of Solids, Dresden 01187, Germany
[2]College of Letters and Science, University of California, Los Angeles, California 90095, USA
[3]Dresden Center for Nanoanalysis (DCN), Center for Advancing Electronics Dresden (CFAED), TUD Dresden University of Technology, Dresden D-01062, Germany
[4]Institute of Metal Research, Chinese Academy of Science, Shenyang, Liaoning 110016, China
[5]Department of Condensed Matter Physics, Weizmann Institute of Science, Rehovot 7610001, Israel
[6]Physics Department, University of Ioannina, Ioannina 45110, Greece

[*] surya.panda@cpfs.mpg.de; edouard.lesne@cpfs.mpg.de; claudia.felser@cpfs.mpg.de

I. Grain analysis using transmission electron microscopy

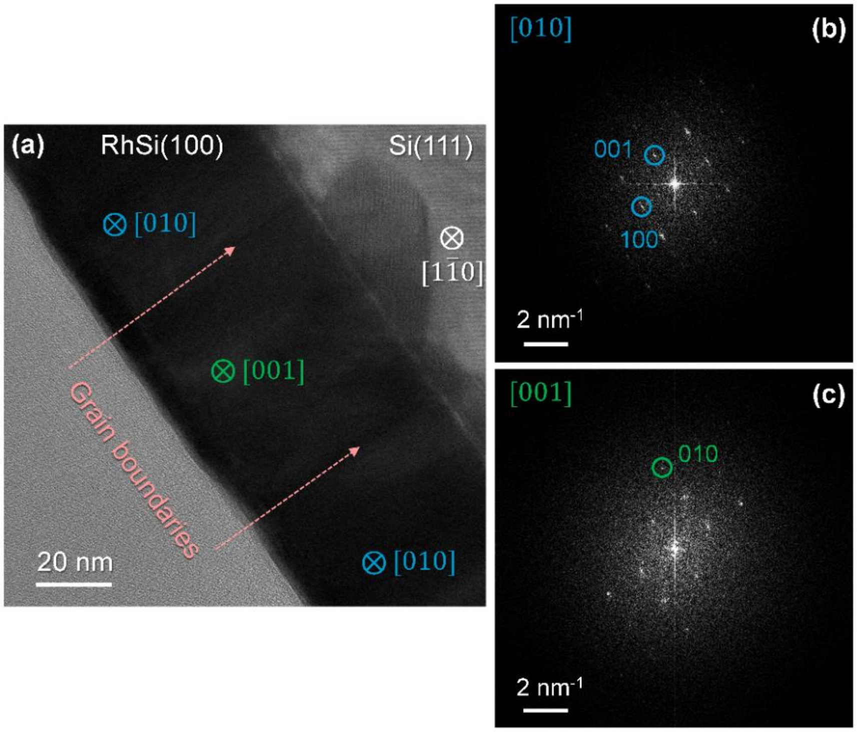

Figure S1: (a) Cross-section HRTEM image of RhSi (same as in Fig. 1e) showing differently oriented crystalline grains and their boundaries (marked by arrows). Local fast Fourier transform showing adjacent grains possessing (b) [010] and (c) [001] in-plane crystallographic orientations

The textured growth of the RhSi film was investigated by HRTEM imaging. Figure S1(a) shows a cross section image of three grains in the RhSi film. All grains grow with the [100] direction parallel to the [111] substrate normal, verified by local FFTs (see Figures S1(b) and S1(c)). The adjacent grains have [010] and [001] zones axis with the (001) and (010) planes parallel to the substrate surface. The grains have a size of roughly 60 nm with straight grain boundaries from the RhSi/Si interface to the RhSi/Py interface.

.

II. Longitudinal resistivity of RhSi crystalline thin films

The temperature-dependent measurements of the longitudinal resistivity (ρ_{xx}) were conducted on thin films of RhSi of various thicknesses. These measurements were performed using a van der Pauw geometry within a PPMS (Physical Property Measurement System) cryostat provided by Quantum Design. RhSi samples capped *in situ* with Si (forming a naturally oxidized ~ 3nm-thick SiO_x capping layer), but without a permalloy overlayer were deposited in order to assess their intrinsic electrical transport properties. The four corners of square-shaped samples were contacted via ultrasonic wedge-bonding technique, using aluminium wires. In Figure S2, we present the longitudinal resistivity versus temperature, $\rho_{xx}(T)$, for RhSi(t)/SiO_x(3 nm) films of various thicknesses. All the films exhibit a metallic behavior, characterized by a continuous decrease in $\rho_{xx}(T)$ as the temperature is lowered from 320 K down to 2 K. The residual resistivity values ($\rho_{xx,0}$), at 2 K, are comprised between 54.6 µΩ.cm for the thicker film and 69 µΩ.cm for the thinner one. The corresponding range of residual resistivity ratios (RRR) is 1.7-2.0. Furthermore, we note that the resistivity did not exhibit a saturation behaviour at low T, implying that within the temperature range under investigation, $\rho_{xx,0}$ was not primarily influenced by extrinsic scattering mechanisms. Specifically, this suggests that factors such as grain boundaries, impurities, interfaces, or crystallographic defects whose presence would typically lead to higher residual resistivity are not the dominant contributors to the low T resistivity in our RhSi films.

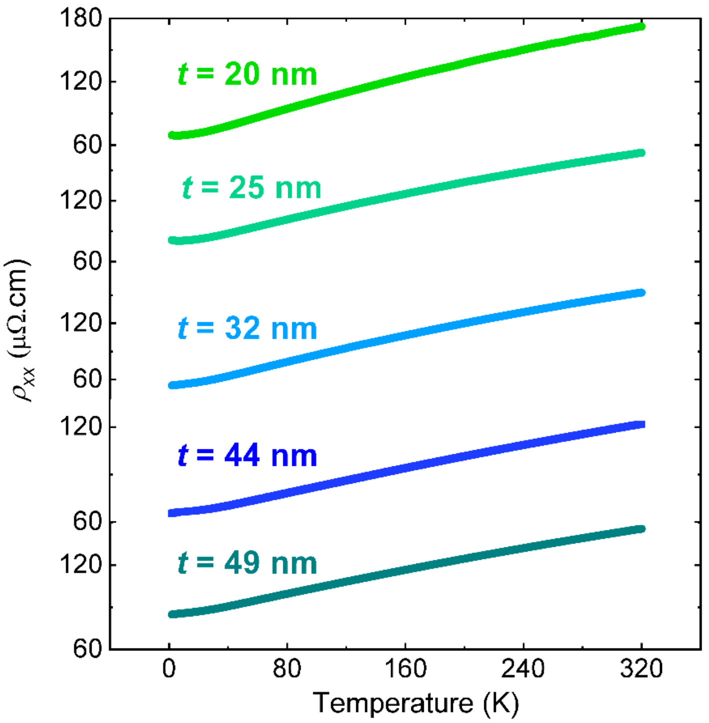

Figure S2: Longitudinal resistivity as a function of temperature, $\rho_{xx}(T)$, for RhSi(t) crystalline thin films (capped with 3 nm-thick SiO_x).

III. Symmetric and antisymmetric components in ISHE measurements

The measured ISHE voltage is plotted against the external magnetic field in Figure S3(a) for the RhSi(49.1 nm)/Py(6.6 nm) sample at 8 GHz frequency. Here, V_{sym} and V_{asym} components are shown by solid red and magenta lines, extracted from the fit using Eq 6. A significantly higher value of V_{sym} in comparison to V_{asym} indicates that spin pumping is the dominant mechanism in the enhancement of α in RhSi/Py heterostructures. The reversal of the sign of V_{ISHE} is observed by reversing the magnetic field (in Figure S3(b)), which excludes that this ISHE signal is produced by a possible thermoelectric effect induced by the ferromagnetic resonance absorption.

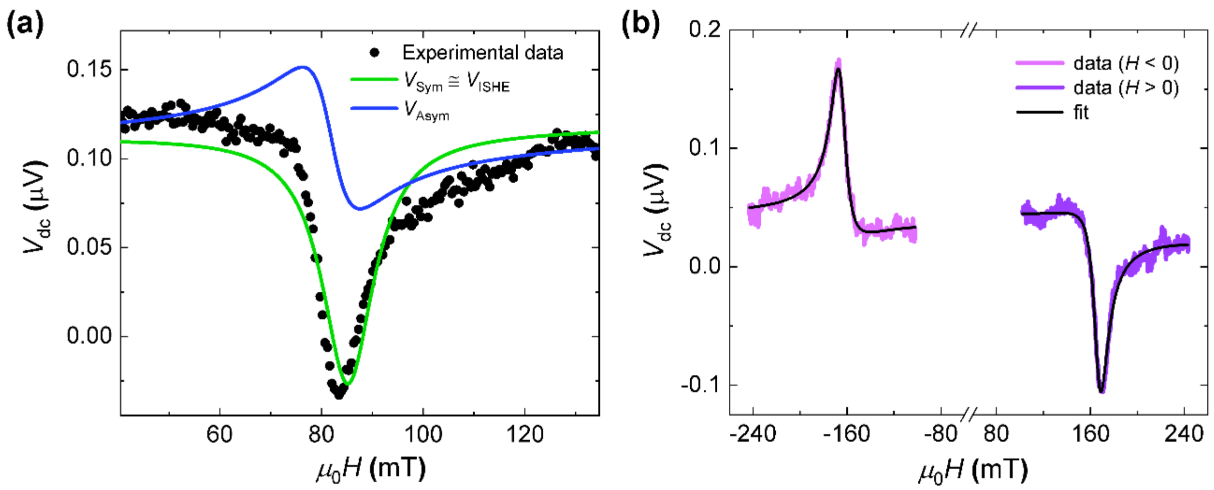

Figure S3: (a) Measured dc-voltage (V_{dc}) *vs.* applied magnetic field for the RhSi(49 nm)/Py(6.6 nm) sample at 8 GHz. The solid green corresponds to the field-symmetric contribution which is identified as the ISHE signal. The solid blue line corresponds to the field-antisymmetric contribution. (b) Measured V_{dc} upon in-plane magnetic field reversal. The solid black lines are the fit using Eq. 6 of the main manuscript.

IV. Temperature- and thickness-dependence of Gilbert damping parameter and saturation magnetization

In Figure S4, we present the modulation in effective saturation magnetization (M_{eff}) and Gilbert damping parameter (α) with the thickness of RhSi and system temperature. Our findings reveal that α maintains a relatively constant across varying RhSi thicknesses, indicating the absence of spin angular momentum backflow through spin pumping. Moreover, this underscores that the spin diffusion length (λ_{sd}) of RhSi is significantly less than 19 nm. Additionally, we observe a monotonic decrease in α with decreasing temperature in Py thin films, as shown in Figure S5(a). However, in RhSi/Py heterostructures, α increases with temperature (as displayed in Figure S5(b)). This temperature dependence of α in magnetic thin films can be modeled as [S1, S2]:

$$\alpha(T) = \alpha_{\text{int}}(T) + \alpha_{\text{SP}}, \tag{S1}$$

$$\text{with } \alpha_{\text{int}}(T) = \alpha_{\text{intra}} \frac{\sigma(T)}{\sigma(300\ K)} + \alpha_{\text{inter}} \frac{\rho(T)}{\rho(300\ K)}. \tag{S2}$$

Here, α_{int} denote the intrinsic Gilbert-like damping of the FM and α_{SP} the additional damping contribution arising from spin pumping into the adjacent NM layer. $\alpha_{\text{intra}}$and α_{inter} relate to the intra-band and inter-band contributions, respectively, which are found to be dependent on the

conductivity (σ) and resistivity (ρ) of the sample. α_{SP} is characteristically inversely proportional to the temperature while the α_{int} can have both direct or inverse dependence on temperature depending upon the leading mechanism of spin-dependent scattering. In the RhSi/Py heterostructures, the increase in α with temperature indicates a dominant intra-band conductivity-like scattering contribution, which is proportional to the momentum relaxation time. Higher temperatures enhance the localization of interactions between magnons and conduction electrons in these heterostructures. In contrast, when the RhSi underlayer is absent, the decrease in α with temperature suggests that inter-band resistivity-like contributions play a predominant role in spin-dependent scattering processes during magnetization dynamics.

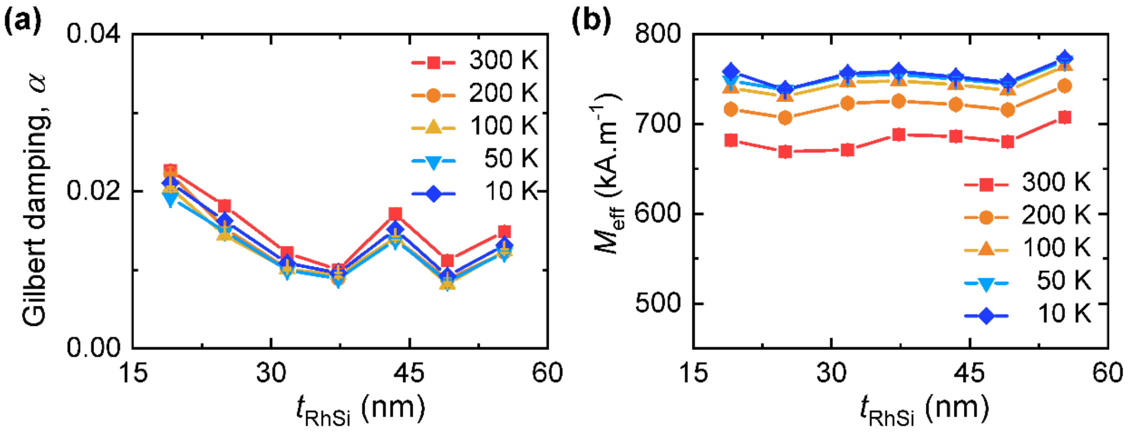

Figure S4: Temperature- and RhSi-thickness-dependent modulation of (a) Gilbert damping parameter α, and (b) effective saturation magnetization M_{eff}.

Furthermore, our observations indicate that M_{eff} remains nearly constant across different RhSi thicknesses (see Figure S4(b)). This implies that interfacial anisotropy remains consistent throughout the range of RhSi thicknesses examined, exerting only a minor influence on the modification of magnetization dynamics within these heterostructures. Both in the presence and absence of RhSi underlayer (shown in Figure S5), M_{eff} decreases monotonically with temperature. This decrease in M_{eff} with temperature can be directly correlated to the Bloch's law ($M_{\mathrm{eff}}(T) = M_{\mathrm{eff}}(0)\left(1 - AT^{3/2}\right)$), where $M_{\mathrm{eff}}(0)$ and B represent the saturation magnetization at T = 0 K and a parameter related to the exchange stiffness constant, respectively [S3].

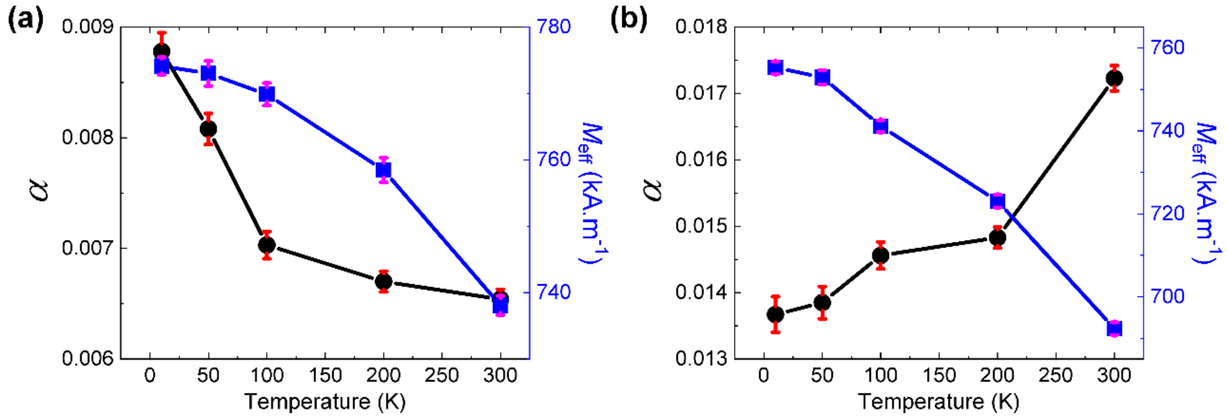

Figure S5: Temperature-dependent variation of Gilbert damping parameter(α) and effective saturation magnetization (M_{eff}) in the (a) Py (6.6 nm) and (b) RhSi(49.1 nm)/Py (6.6 nm) thin films.

V. Permalloy thickness-dependent magnetization dynamics

Within an NM/FM heterostructure, apart from the influence of spin pumping, there exists a distinct probability for the dissipation of spin angular momentum, originating from interfacial depolarization and surface irregularities. These phenomena are commonly referred to as "spin-memory loss" (SML) and "two-magnon scattering" (TMS). In the mechanism of SML, the dissipation of spin angular momentum occurs when the atomic lattice at the interface acts as a reservoir for spin states. This may result from the magnetic proximity effect or interfacial spin-orbit scattering, leading to the transfer of spin polarization to the atomic lattice. TMS, on the other hand, occurs when a uniform FMR mode is disrupted, giving rise to the creation of a degenerate magnon with a different wave vector. The non-conservation of momentum in this process can be explained by considering a pseudo-momentum derived from internal field irregularities or secondary scattering events. Both SML and TMS can significantly contribute to the enhancement of the α. In the presence of these effects, the modulation of α can be approximated as follows [S4]:

$$\Delta\alpha = g\mu_B \frac{g_{\uparrow\downarrow}^{\mathrm{eff}} + g_{\mathrm{SML}}}{4\pi t_{\mathrm{Py}} M_s} + \beta_{\mathrm{TMS}} {t_{\mathrm{Py}}}^{-2} \qquad \text{(S3)}$$

Here, g_{SML} represents the "spin-memory loss conductance" and β_{TMS} is the "TMS coefficient". We have employed this equation to analyze the dependence of α on the inverse of Py layer thickness to discern the individual influences of the SML, TMS, and spin pumping (SP). For this study, we utilized samples with and without 43.5 nm RhSi underlayer where Py thickness is varied from 6.6 nm to 30 nm.

Figures S6(a) and S6(b) depict the Py-thickness-dependent variations of α at 300K and 10 K system temperature. In the absence of RhSi, α remains small and independent of Py thickness. However, in the presence of RhSi, α exhibits a linear increase with the inverse of Py thickness. Table S1 presents the values of β_{TMS}, g_{SML}, and $g_{\uparrow\downarrow}^{\mathrm{eff}}$ obtained through the fitting of Py-thickness-dependent α using Equation (S3). It shows that, as the temperature decreases, the relative extrinsic contributions diminish significantly. Figure S6(c) shows the Py-thickness-dependent relative contributions of extrinsic TMS and SML effects to α at 300K. It is evident that the extrinsic contribution is well below 10 % of the damping modulation for all the RhSi/Py samples. At lower thickness, this contribution becomes slightly higher but remains much lower than the intrinsic SP contribution. We have extracted the interfacial magnetic anisotropy energy density (K_s) which is an indicator of the strength of the interfacial spin-orbit coupling (SOC) by fitting the Py-thickness-dependent M_{eff} (see Figure S6(d)) with the formula [S5]:

$$4\pi M_{\mathrm{eff}} = 4\pi M_{\mathrm{S}} - \frac{2K_{\mathrm{S}}}{M_s t_{\mathrm{Py}}} \tag{S4}$$

From the fit, we have extracted the values of K_s to be 77 $\times 10^{-5}$ J/m^2 and 43 $\times 10^{-5}$ J/m^2 in the presence and absence of the RhSi underlayer, respectively. This increase in K_{s} value in the presence of RhSi is an indication of the increase in interfacial SOC strength [S5]. The saturation magnetization (M_s) also decreases from 848 kA/m in the absence of RhSi to 813 kA.m^{-1} in the presence of RhSi underlayer. Table S1 presents the temperature-dependent values for K_{s} and M_{S}. This illustrates that, in accordance with Bloch's law, as the temperature decreases, M_{S} undergoes an increase, while K_{s} demonstrates a minor decrease, signifying a subtle enhancement in interfacial SOC strength.

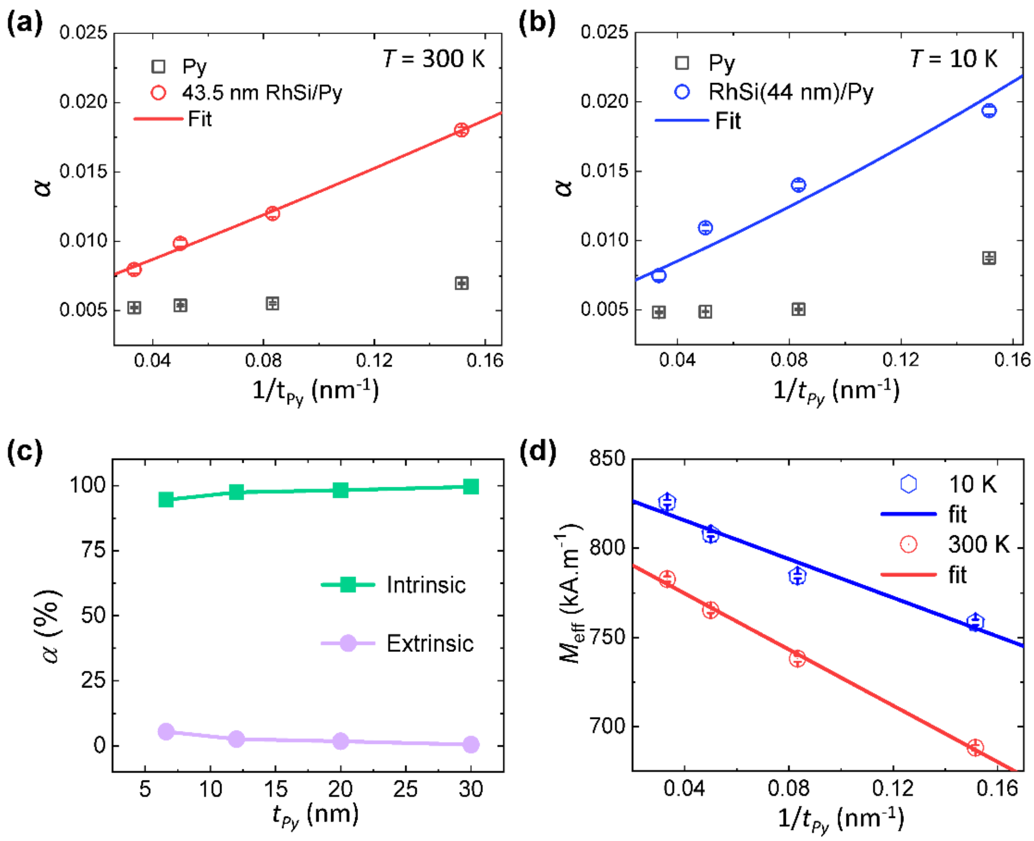

Figure S6: Enhancement of effective Gilbert damping parameter with the inverse of permalloy thickness at (a) 300 K and (b) 10 K. The red solid lines represent the fits using Equation (S3). (c) Percentage contribution in the modulation of damping from extrinsic spin memory loss and two-magnon scattering across the thickness range of the permalloy layer. (d) Variation of M_{eff} with $1/t_{Py}$. Symbols are experimental data points and solid red lines are fits using Equation (S4).

Table S1: Temperature-dependent spin-mixing conductance ($g_{\uparrow\downarrow}$), spin-memory loss (SML) conductance (g_{SML}), two-magnon scattering (TMS) coefficient (β_{TMS}), saturation magnetization (M_s), and interfacial magnetic anisotropy energy density (K_s) in the RhSi(43.5 nm)/Py heterostructures.

Temperature (K)	β_{TMS} (nm^2)	g_{SML} (nm^{-2})	$g_{\uparrow\downarrow}^{eff}$ (nm^{-2})	M_s (kA.m^{-1})	K_s (10^{-5} J/m^2)
300	0.42	1.47	38.7	813.1	43
10	0.18	1.01	25.3	844.2	32

Supplementary References

[S1] B. Heinrich, D. J. Meredith, and J. F. Cochran, J. Appl. Phys. **50**, 7726–7728 (1979).

[S2] B. Khodadadi, A. Rai, A.Sapkota, A. Srivastava, B. Nepal, Y. Lim, D. A. Smith, C. Mewes, S. Budhathoki, A. J. Hauser, M. Gao, J. Li, D. D. Viehland, Z. Jiang, J. J. Heremans, P. V. Balachandran, T. Mewes, and S. Emori, Phys. Rev. Lett. **124**, 157201 (2020).

[S3] Y. Zhou, R. Mansell, S. Valencia, F. Kronast and S. van Dijken, Phys. Rev. B **101**, 054433 (2020).

[S4] L. Zhu, D. C. Ralph and R. A. Buhrman, Phys. Rev. Lett. **123**, 057203 (2019).

[S5] G. Wu, Y. Ren, X. He, Y. Zhang, H. Xue, Z. Ji, Q. Y. Jin and Z. Zhang, Phys. Rev. Appl. **13**, 024027 (2020).
